# Cloud Computing Mobile Application for Remote Monitoring of Bell’s Palsy

**DOI:** 10.1007/s10916-020-01605-7

**Published:** 2020-07-29

**Authors:** P Watts, P Breedon, C Nduka, C Neville, V Venables, S Clarke

**Affiliations:** 1 Medical Engineering Design Research Group, Nottingham Trent University, Nottingham, UK; 2 Queen Victoria Hospital, NHS Trust, East Grinstead, West Sussex UK; 3 Emteq Ltd, Sussex Innovation Centre, Brighton, UK

**Keywords:** Cloud computing, Mobile application, Bell’s palsy, Healthcare solution, Wearable technology, System design

## Abstract

Mobile applications provide the healthcare industry with a means of connecting with patients in their own home utilizing their own personal mobile devices such as tablets and phones. This allows therapists to monitor the progress of people under their care from a remote location and all with the added benefit that patients are familiar with their own mobile devices; thereby reducing the time required to train patients with the new technology. There is also the added benefit to the health service that there is no additional cost required to purchase devices for use. The Facial Remote Activity Monitoring Eyewear (FRAME) mobile application and web service framework has been designed to work on the IOS and android platforms, the two most commonly used today. Results: The system utilizes secure cloud based data storage to collect, analyse and store data, this allows for near real time, secure access remotely by therapists to monitor their patients and intervene when required. The underlying framework has been designed to be secure, anonymous and flexible to ensure compliance with the data protection act and the latest General Data Protection Regulation (GDPR); this new standard came into effect in April 2018 and replaces the Data Protection Act in the UK and Europe.

## Introduction

### Background

Bell’s palsy represents 60% of all facial palsy cases current in the UK, with up to 25,640 new cases in the UK annually [1]. The annual incidence rate within the UK population is estimated to be 20 per 100,000 and an estimated 1 in 60 people are often left with a permanent disability with an estimated 100,000 people living in the UK with permanent facial palsy’s [2]. Bell’s palsy has a significant impact on patients’ lives affecting their facial function, appearance and communication. This often affects a patient’s ability to speak, eat without dribbling or blink and is shown to have a significant physiological impact on patients leading to conditions such as depression and anxiety [3, 4].

Facial muscles are different to other skeletal muscles in that they have poor proprioceptive feedback. Most people are unable to control individual facial muscles, such as raising alternating eyebrows. This is by design; the face’s role is that of a method to transact trusted interactions, which is aided by limited conscious control of expressions.

Following injury to the facial nerve, the degree of recovery depends on the severity of the injury which is typically classified by a grading system such as the House Blackman Scale, or the Sunnybrook Facial Grading System [5]. Studies show that early intervention using specialized exercises has a significant impact on improving the patient’s condition [6]. The lack of proprioception makes recovery particularly difficult as patients are usually unaware of abnormal movements their faces exhibit during daily activities. Without additional feedback their facial function may worsen and potentially develop into permanent abnormal movements.

Biofeedback for facial paralysis is well established in specialist centres [7, 8, 9, 10], however, intermittent biofeedback is of limited value. Mobile technology providing real-time feedback and guidance to patients and therapists promises to significantly improve in the rehabilitation of facial paralysis [11].

### Related works

The research sector contains many existing applications and designs for remote home healthcare and throughout the project the team considered the research in its approach to the design and implementation of the FRAME project.

The CARA (Context-Aware Real-time Assistant) project led by Bingchuan Yuan at the University College Cork [12] researched the utilization of wireless remote monitoring of patients via a web browser to allow a healthcare professional or carer to monitor their patients remotely and from any web enabled device.

A team at Edinburgh university led by E Ekonomou in 2011 presented a project on an Integrated Cloud-based Healthcare Infrastructure [13] (DACAR) [X2], this project prioritized high levels of security and privacy whilst utilizing a secure cloud based environment. This is a key priority in the development of any cloud based solution like the FRAME project.

There are a number of related research projects looking into the use of Electromyography (EMG) to detect the movement of facial muscles, a paper by Jun-Wen Tan from the University of Ulm in 2012 investigated the use of EMG to detect the activity over two key muscles in the face to identify various emotional responses [14]. Research by A Van Boxtel at Tilburg University [15] investigated the location of EMG sensors and the methodological aspects of recording the facial EMG signals to identify movements in the key facial muscles.

### Overview

**Fig. 1:**
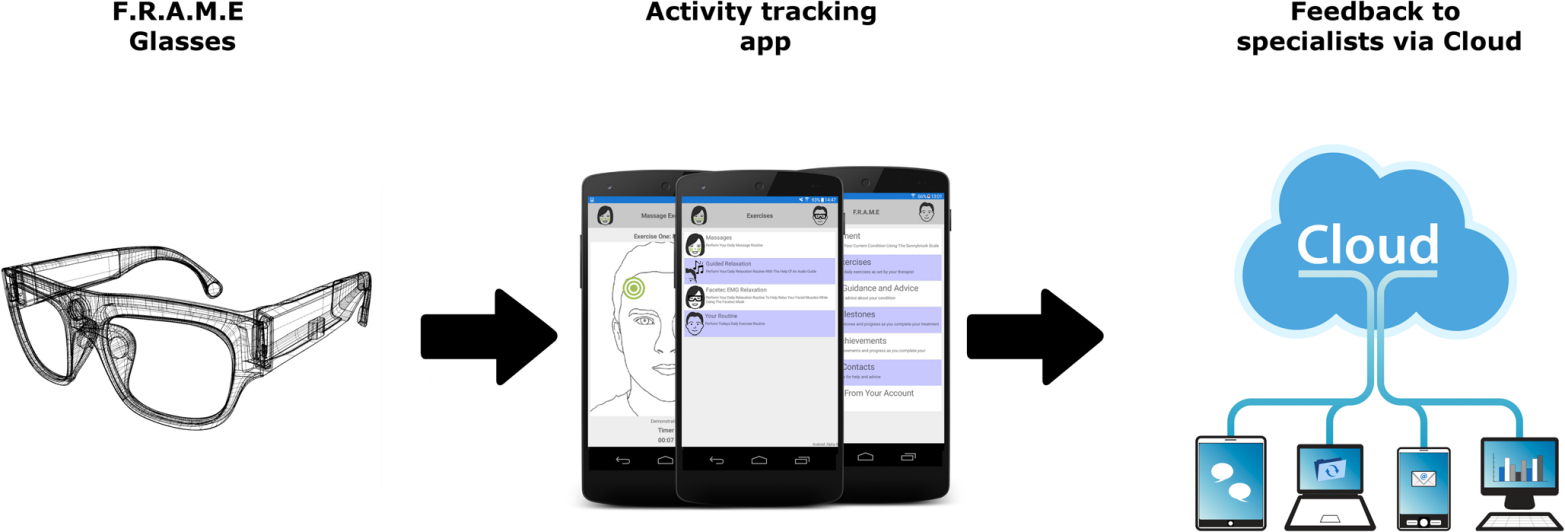
The FRAME system overview

The FRAME project required the development of an online cloud based mobile solution for the treatment and monitoring of patients suffering from Bell’s palsy, the project consists of three key parts all of which make up a framework that comes together to form the final end user product (Fig. 1).

The Facial Remote Activity Monitoring Eyewear (F.R.A.M.E) mobile application is an application designed to work on IOS and Android for the remote care of individuals during their treatment. The application contains information and exercises to aid in the patient’s rehabilitation from Bell’s palsy.

An online web application provides a cloud based Representational State Transfer (REST) application-programming interface (API) hosted on the Microsoft Azure cloud platform for secure transmission of data across multiple devices.

A therapist console built into the web application provides access for permitted therapists (via a web browser) to access patient data remotely. This allows for the remote monitoring of patients progress over a period of weeks, months or years.

The mobile interfaces with two wearable hardware devices developed as part of the FRAME project via Bluetooth Low Energy (BLE) to provide sensor feedback from the patient whilst they are performing exercises as instructed by the mobile application.

### Design requirements

Design of the system is split into three key areas to ensure that the developed system addresses the needs of the patients and therapist whilst also ensuring the system complied with security requirements and GDPR regulations. The first area (Table 1) addresses the patient requirements (PR) of the system, these requirements focus on the functionality within the patient facing mobile application.

**Table 1:** Patient requirements (PR)

PR1	Mobile application for use on personal mobile devices (Apple/Android)
PR2	Ability to use multiple devices throughout treatment such as multiple phones or tablets
PR3	To provide information and advice on living with Bell’s palsy.
PR4	Provide guidance on daily exercises and stretches
PR5	Provide mirror therapy within the mobile application
PR6	Ability to perform exercises daily when it suits them so as to fit in with other daily activities
PR7	Visual guidance with feedback for muscle relaxation whilst using the EMG mask
PR8	Visual guidance with feedback whilst performing a gentle balanced smile with the EMG mask

The second area (Table 2) identified addresses the Therapist Requirements (TR) and focuses on the web console and the ability for therapists to monitor their patients throughout treatment (TR2, TR6). Therapists highlighted the need for information about Bell’s palsy to be readily available to patients within the mobile application (TR4, TR5).

**Table 2:** Therapist requirements (TR)

TR1	Low cost solution for the NHS
TR2	Ability to see patient’s activity and adherence during treatment
TR3	Access to patient data from multiple devices/computers within the NHS
TR4	A digital and easy to find resource to provide NHS information/advice on living with Bell’s palsy.
TR5	Provide easy access to existing video content for managing Bell’s palsy
TR6	Provide access to data from EMG mask exercises

The third area (Table 3) addresses the System Requirements (SR) to ensure data is secured and access is only granted to authorised personnel (SR1, SR2). It also ensures that the designed system complies with data protection and GDPR regulations (SR3).

**Table 3:** System requirements (SR)

SR1	Secure login with encryption of username and passwords
SR2	Secure access to data for authorized personnel only
SR3	Anonymous data storage
SR4	24/7 Access to data and services

## System framework

The F.R.A.M.E application framework consists of a group of system modules running on multiple platforms, all connected to one another to form the core of the F.R.A.M.E system framework. Each individual system module works closely with the other system modules to create a comprehensive, flexible and secure solution.

### Mobile application

The mobile application is designed and developed to work on IOS and Android (PR1, TR1), allowing deployment onto the majority of mobile devices currently on the market; also allowing deployment onto personal devices of the majority of patients receiving treatment (TR1). The mobile application utilizes the Xamarin Cross Platform programming language to allow for simultaneous development of the application for both platforms, sharing common code between the two platforms. This shared code speeds up development by reducing the time to develop separate solutions and thereby reduces costs of overall development.

**Fig. 2:**
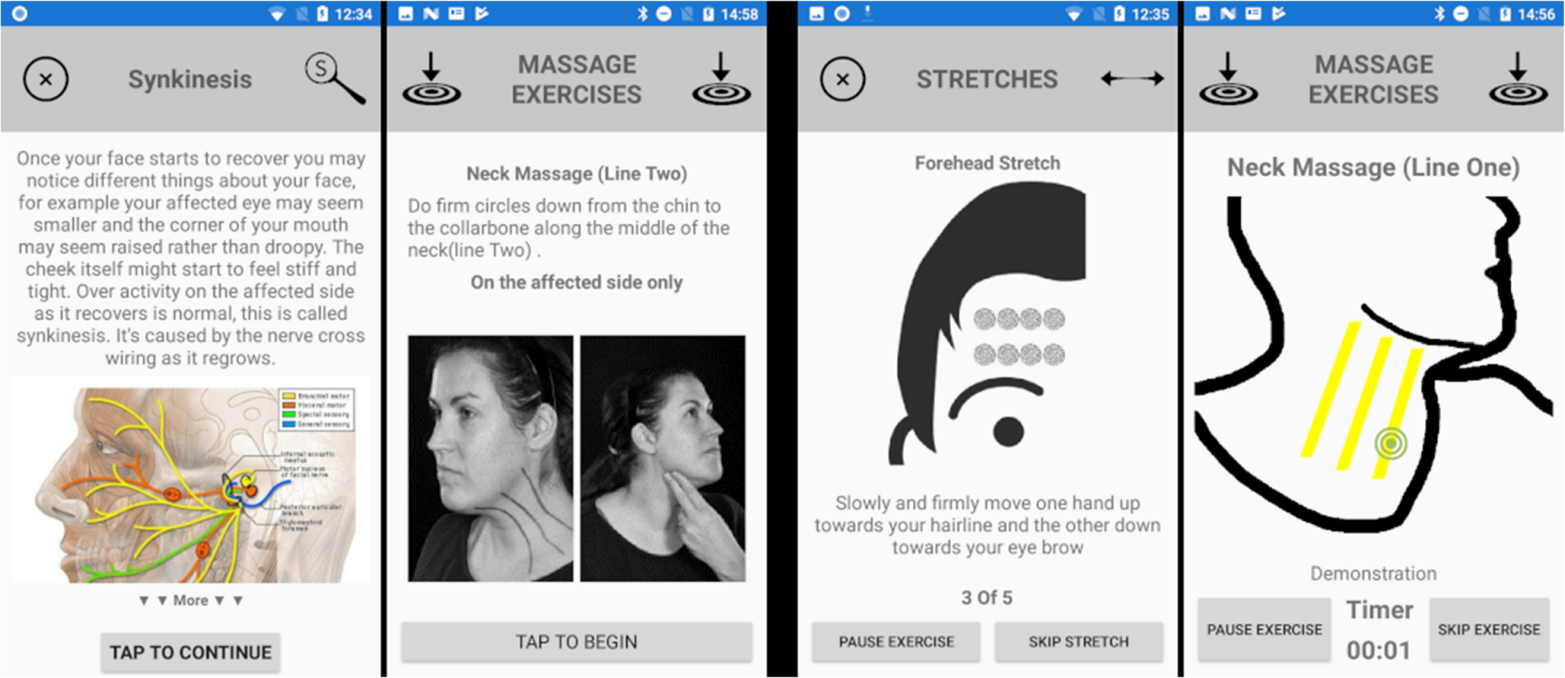
Screenshots from the F.R.A.M.E App. From left to right, patient information, exercise instructions, animated stretch and animated massage exercises

The aim of the application is to assist patients by providing essential information and advice helping them manage their condition daily. Information has been taken from current NHS guidance handouts and DVD video resources (TR5) and has been integrated into categorized sections of the application (PR3, TR4). The application provides animated guidance and timings for performing daily stretch and massage exercises, aiding patient’s recovery and rehabilitation. Massage and stretch guidance is provided by a set sequence of exercises with accompanying animations all timed to be copied in line with current NHS guidelines (PR4, TR4). An example of screens from the app can be seen in Fig. 2. The patient can perform these exercises at any time of day (PR6) with the mobile application sending usage statistics to the cloud server for observation and monitoring by the therapist (TR2).

The mobile application connects to the F.R.A.M.E server via any available internet connection on the device, allowing for the retrieval and transmission of data between the server and client. Data is stored on the cloud server and not the device; this allows a patient to swap seamlessly between multiple devices if required (PR2). This is an important design aspect when developing for applications designed for use over extended periods, users can own multiple devices and devices can fail and/or be replaced during treatment. For security, the application requires entry of a secure username and password chosen by the user during registration.

### F.R.A.M.E EMG goggles

The F.R.A.M.E project involved the development a hardware device to assist in the rehabilitation of patients and to provide feedback to the therapist throughout treatment [9, 10]. The device connects to the mobile application via Bluetooth Low Energy (BLE).

**Fig. 3:**
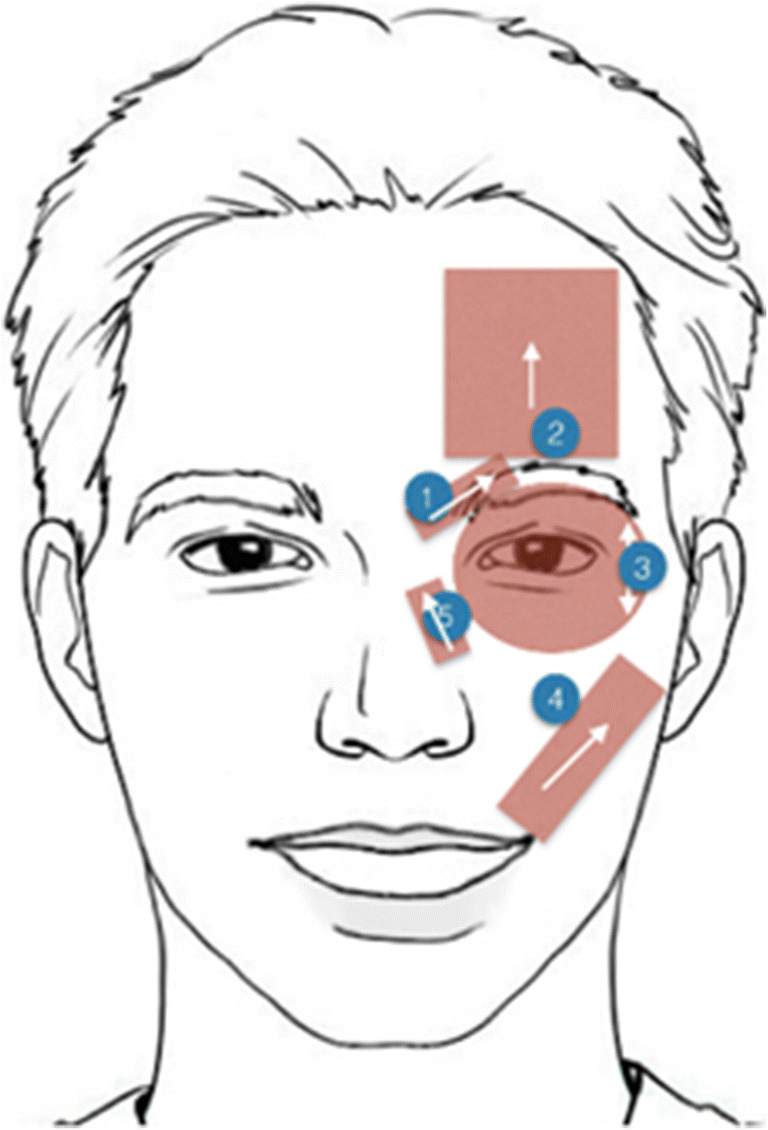
Right side of the EMG mask sensor placements with corresponding movement indicators for each sensor

The hardware takes the format of a pair of goggles with integrated Electromyography (EMG) sensors places at 10 key locations around the patients face to allow the collection and monitoring of key facial muscles responsible for expressions (Fig.3) [9, 10]. EMG data is streamed to the mobile application via BLE where it is stored on the device prior to being uploaded to the server using a RESTful API.

**Fig. 4:**
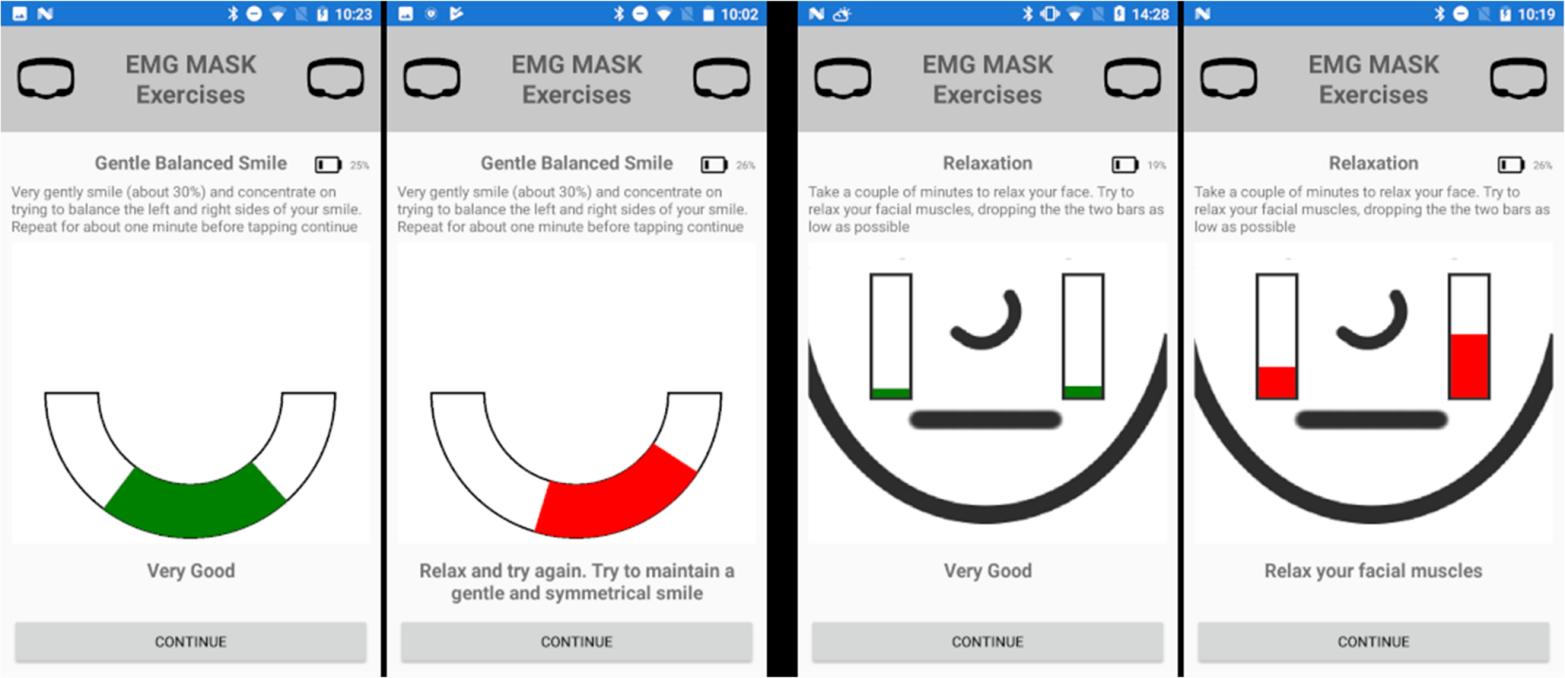
EMG relaxation and gentle smile exercise within the mobile application

The application currently consists of an exercise routine containing two exercises chosen to best test the mask and its usability during real world conditions. Patients facial muscles need to be relaxed before performing any voluntary facial expression to prevent strain and further damage, with this in mind the first exercise is designed to help the user relax their facial muscles (PR7) and to make them aware of tension in any of their facial muscles (Fig. 4, right). The second exercise instructs the user to perform a gentle balanced smile (PR8) whilst focusing on achieving an even and balance smile between the left and right side of their facial muscles (Fig. 4, left). Should the user exhibit an unbalanced smile the EMG sensors will pick up on the variation and provide user feedback on the mobile application with on screen instructions to the user to relax and gently try again. The smile exercise was chosen by therapists on the team to best validate the mask with a vision to expand the number of exercises once validation has been completed.

### Cloud web application

The web application is deployed on the Microsoft Azure cloud platform and is written in C# using the MVC 5 framework. The purpose of the web application is to provide the implementation of the RESTful API for data transmission and to provide the therapist and administrator web portals accessible via any web browser. The web application utilises a secure Azure hosted MSSQL database for storage of linked data sets and blob storage for the storage of larger chunks of data such as files and images.

### RESTful API

The RESTful [16] web API has been developed to communicate with the mobile application using JavaScript Object Notation (JSON) [17]. This is a lightweight machine-readable data interchange format. Data is passed between the mobile application and web application via a Secure Sockets Layer (SSL) [18] connection utilising Hypertext Transfer Protocol (HTTP) [19] POST commands. All content is encrypted as part of the SSL layer in order to prevent the data being intercepted, read or modified during transmission.

### Therapist portal

**Fig. 5:**
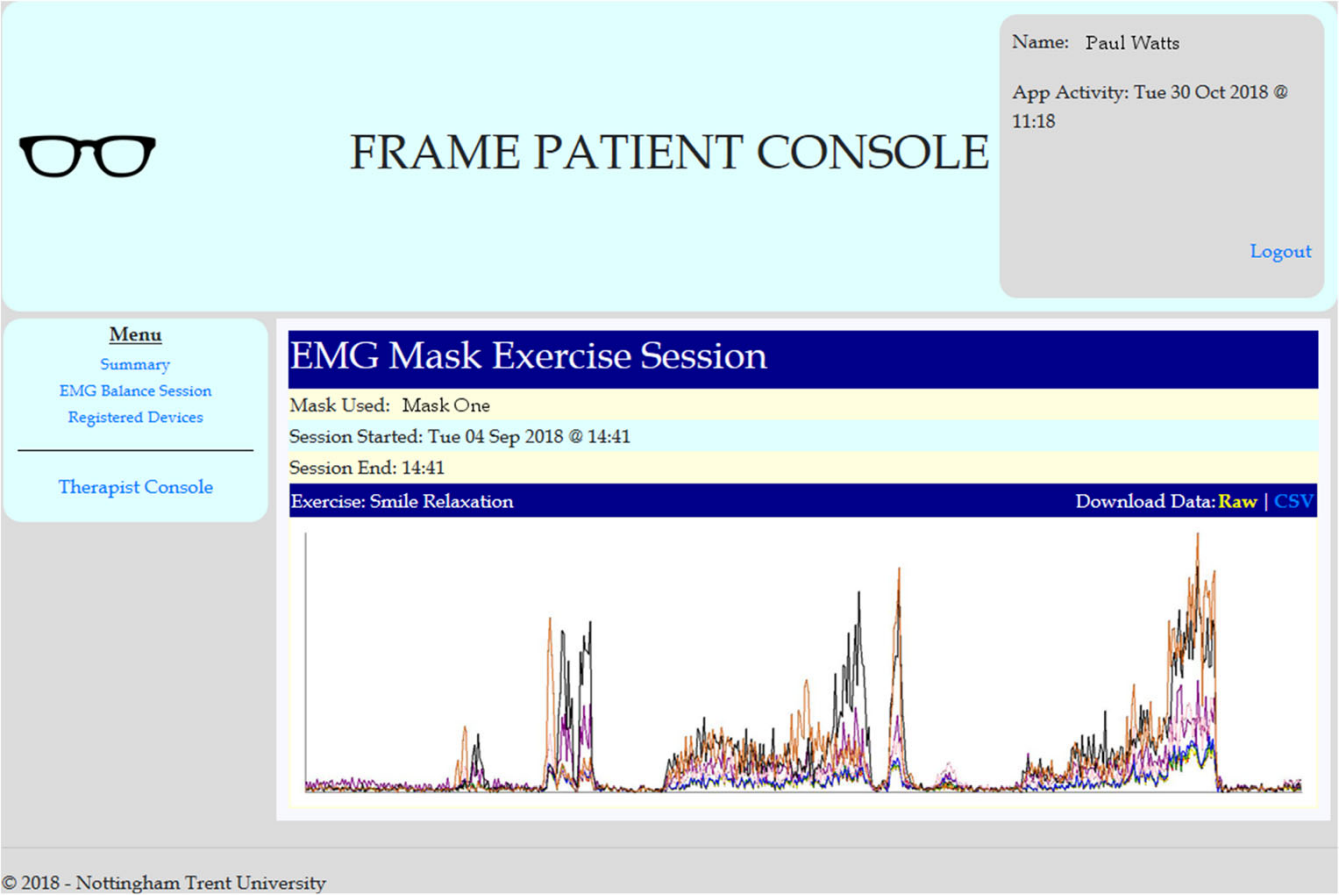
Example of patient console as seen by the logged in therapist

A therapist portal is built into the web application as a website and is accessed from any web browser on a mobile device, Windows computer or Mac computer (TR3). The website provides a secure interface for registered therapists to view the progress of patients under their care. Access to the website requires therapists to login using a valid username and password (SR2). The main console allows the therapist to see a quick overview of patients under their care and they can see recent activity for each patient with inactive patients clearly indicated (TR2). The purpose of this view is to highlight patients who are not currently active in their treatment allowing the therapist to address key concerns and intervene should they see fit. The therapist has the option to see more details about each patient via a ‘Patient Console’ link. The patient console is designed to show information relating to an individual patient and shows the therapist the patient’s current activity level and data collected from exercises performed by the patient during treatment (TR2). The therapist can access and analyze the data when needed (TR6). A visual representation of the EMG data collected by the mobile application from mask device is drawn as a graph showing the muscle activity of the patient throughout the exercise. Figure 5 shows an example of the patient console within the therapist portal.

The console also provides access to this data in the form of download links for files in raw or Comma-separated values (CSV). CSV files can be opened in many common software packages such as Excel and allows for further analysis by the therapist should it be needed (TR6). The contents of these files is anonymized for security and data protection purposes.

## Security

### Account login

In order for a patient to access the system, they are required to login via the mobile application using a username and password. The login system has been developed using a token-based authentication system to reduce the possibility of data being stolen and usernames and passwords being compromised (SR1).

**Fig. 6:**
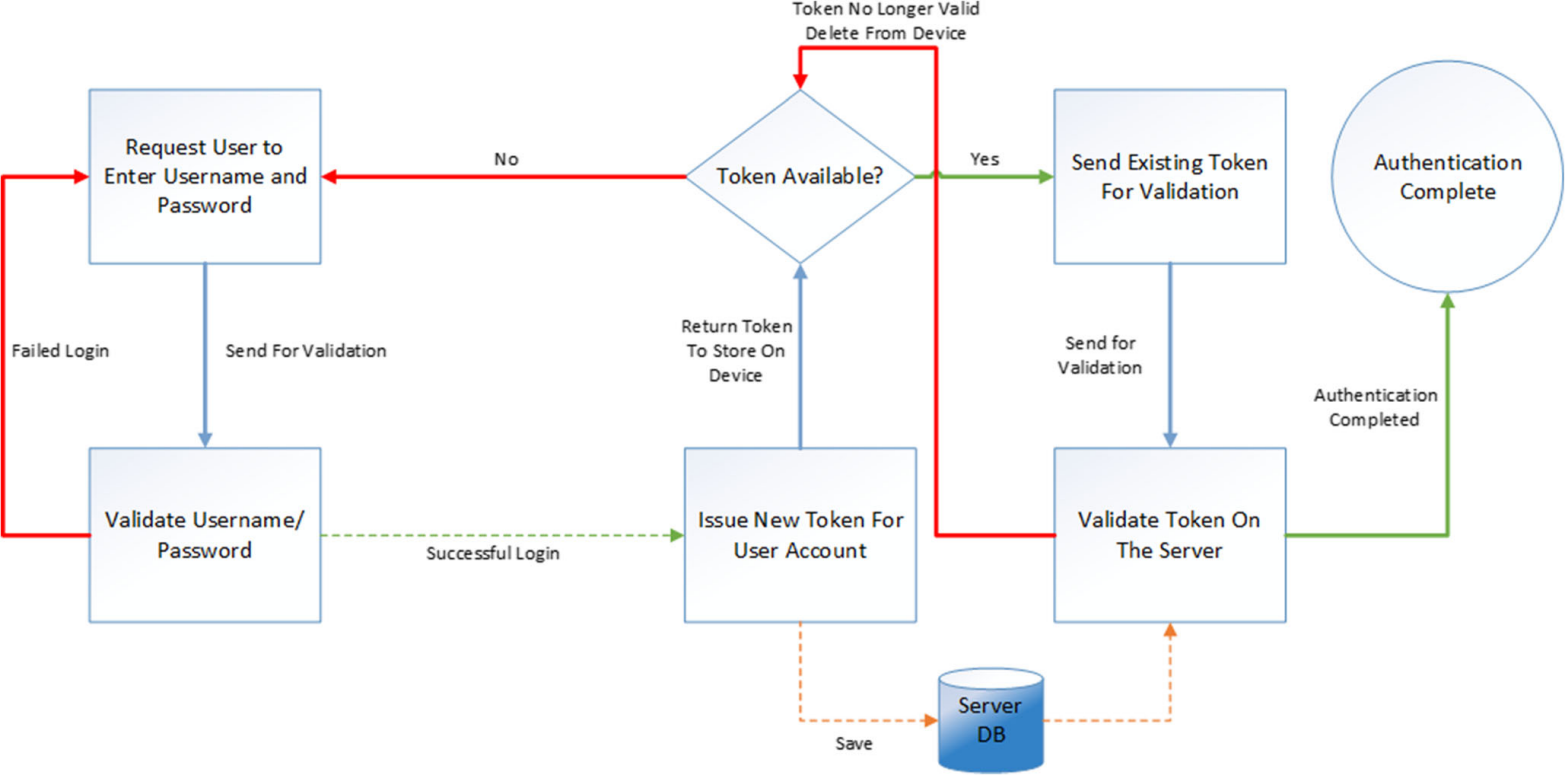
Token Authentication Security Flow

Token based authentication works by registering a device to the user account on the server system and issuing a unique and randomized token. The first time the application is started the user is asked to enter their username and password, this is encrypted using AesCbcPkcs7 encryption before being transmitted to the server via the API, utilizing a secure SSL encrypted Post command. The username and password are validated on the server and a Globally Unique Identifier (GUID) is assigned as a unique token to the user account and device id. This token is sent back to the mobile application and the application stores the token, at no point is the password stored on the mobile device. All transactions made to the server after authentication will utilize the token along with the device id. This removes the need to pass the username and password between the device and server system during each API request and removes the need to store the password on the devices itself. The full process is shown in Fig. 6 below.

### Data access

For security reasons the web portal also uses the token authentication to validate the therapist is logged in before streaming any images or files to the end users browser (SR2). Files are not directly available and cannot be downloaded via a standard URL request; the web browser requests the image or file through a secure POST command, the user’s login is validated and the web server accesses the file securely before forwarding data to the browser through a secure SSL encrypted connection.

### Image access and storage

To facilitate the secure storage of images on the server, encryption was added to all images uploaded from the application. Images are encrypted using Rijndael encryption [20] utilizing randomized keys known only to the server. Encryption keys are stored in a secure table not accessible outside of the servers secure network and are stored using a randomized ID to obfuscate the image within the database. Encrypted image data is then stored in secure cloud storage located inside the isolated private network accessible only by the web application. Additionally all data stored within Azure Cloud Storage is itself encrypted by the Azure service using 256 bit AES encryption [21].

**Fig. 7:**
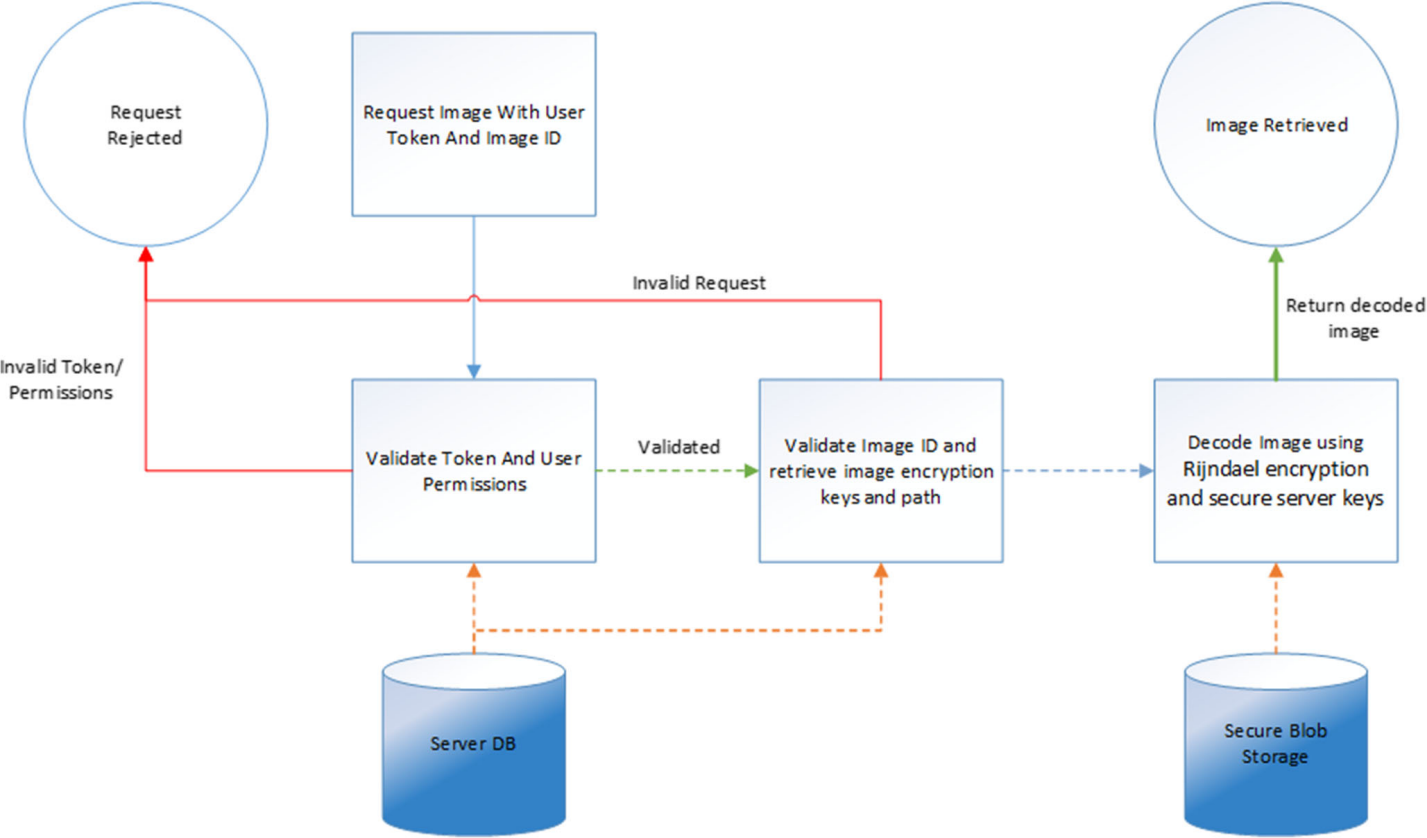
Image Request Data Decryption Flow

To access the image a request needs to be made using a valid token and the image id. Validation is made on the server to ensure the user has permission to access the image before the image is decoded using the secure database keys and returned as a decoded image for use in the application or web browser view. (Fig. 7).

### GDPR and anonymizing patient data

Throughout development of the project, the team carefully considered security and compliance with data privacy and the anonymity of registered patients. In April 2018, Europe introduced the new General Data Protection Regulation (GDPR) rules to replace the data protection act. These rules protect members of the public from having personal data used without their explicit consent and restricts the capability of companies to store personal data without consent from the user.

It was identified during development that the system could function without the need to store personal details that would allow the identification of an individual on the system. The system has no need to know the identity, age or gender of any person using the application. This has no impact on the outcome of the treatment. Using a registration system requiring only a username and password allows user data to be collected, stored and processed anonymously without knowing the identity of the account holder.

Therapists can track a user’s progress through the anonymous usernames displayed on the therapist console. If desired, the therapist can seek verbal permission from an individual to disclose their username, which would provide consent for the individual patient to be identified by the therapist. This information is beyond the scope of the server system as no identifiable data is stored and will be the responsibility of the therapist to ensure GDPR compliance is adhered too.

## Conclusion

Personal mobile devices and cross platform application development tools allow for the development of low cost medical intervention applications to aid and assist a wide range of patients suffering long term medical conditions. Office for National Statistics mobile phone data 2018 to 2017 reports indicate that there are over 83 million mobile phone subscriptions with 93% of adults owning such a device [22]. The healthcare sector would benefit greatly by tapping into this resource to both cut costs and use these platforms to provide expert advice to members of the public through official and endorsed mobile applications.

The F.R.A.M.E application has been designed and developed to include the latest advice from healthcare specialists working within the NHS. This help and advice would normally be distributed using paper based documents within the clinic and these handouts can often be misplaced or forgotten by the patient once they leave the clinic. The F.R.A.M.E mobile application includes this information and is a single point of reference whilst also providing visual guidance on exercises and stretches. These exercises guide correct compliance and adherence to the exercise routines to improve clinical outcomes and benefits of the treatment. Information can be updated regularly to provide the latest advice and information from experts within the NHS through application updates and updates to the information stored on the cloud platform.

Utilization of cloud based web applications allows for remote monitoring of patients and is available 24 h a day, 7 days a week, and 365 days a year. Allowing patients to perform their exercises when it suites them and providing information and advice as and when they need it. Patients adhering to these exercise routines and advice will receive the best treatment available whilst being able to carry on with their everyday lives uninterrupted.

A therapist console has been integrated into the solution to allow therapists to regulate their workload and efficiently manage their time, focusing on patients with the greatest need as identified through the therapist online portal. Therapists can view the activity of their patients at any time, without interrupting the patient’s everyday activities, allowing for a passive treatment method with intervention only supplied as and when needed.

When designing application frameworks for use in the healthcare sector careful consideration is needed to ensure GDPR compliance. GDPR data protection laws require that it is necessary to collect only user details that is essential to the treatment process. F.R.A.M.E demonstrates that it is feasible for an online treatment system to be designed, developed and deployed with minimal personally identifiable data being required.

The team envisions further developing the solution to include more exercises into the routine and to increase the monitoring functionality of the cloud solution to ensure that the best treatment is achieved. Further refinement in the design of the hardware will improve the sensitivity of the mask and allow for compatibility with different facial morphologies. A second hardware device is currently in development to aid patients at the next stage of treatment. Patients suffering with Synkinesis will be able to wear a pair of electronic glasses which alerts them to their condition to allow for voluntary changes in their behaviour.
